# Nitrogen Fixation in Cereals

**DOI:** 10.3389/fmicb.2018.01794

**Published:** 2018-08-09

**Authors:** Mónica Rosenblueth, Ernesto Ormeño-Orrillo, Aline López-López, Marco A. Rogel, Blanca Jazmín Reyes-Hernández, Julio C. Martínez-Romero, Pallavolu M. Reddy, Esperanza Martínez-Romero

**Affiliations:** ^1^Center for Genomic Sciences, Universidad Nacional Autónoma de México, Cuernavaca, Mexico; ^2^Laboratorio de Ecología Microbiana y Biotecnología, Departamento de Biología, Facultad de Ciencias, Universidad Nacional Agraria La Molina, Lima, Peru; ^3^Centro de Investigación en Genética y Ambiente, Universidad Autónoma de Tlaxcala, Tlaxcala, Mexico; ^4^The Energy and Resources Institute, India Habitat Centre, New Delhi, India

**Keywords:** rice, corn, wheat, sorghum, diazotrophic bacteria, root colonization, *Rhizobium*, Burkholderia

## Abstract

Cereals such as maize, rice, wheat and sorghum are the most important crops for human nutrition. Like other plants, cereals associate with diverse bacteria (including nitrogen-fixing bacteria called diazotrophs) and fungi. As large amounts of chemical fertilizers are used in cereals, it has always been desirable to promote biological nitrogen fixation in such crops. The quest for nitrogen fixation in cereals started long ago with the isolation of nitrogen-fixing bacteria from different plants. The sources of diazotrophs in cereals may be seeds, soils, and even irrigation water and diazotrophs have been found on roots or as endophytes. Recently, culture-independent molecular approaches have revealed that some rhizobia are found in cereal plants and that bacterial nitrogenase genes are expressed in plants. Since the levels of nitrogen-fixation attained with nitrogen-fixing bacteria in cereals are not high enough to support the plant’s needs and never as good as those obtained with chemical fertilizers or with rhizobium in symbiosis with legumes, it has been the aim of different studies to increase nitrogen-fixation in cereals. In many cases, these efforts have not been successful. However, new diazotroph mutants with enhanced capabilities to excrete ammonium are being successfully used to promote plant growth as commensal bacteria. In addition, there are ambitious projects supported by different funding agencies that are trying to genetically modify maize and other cereals to enhance diazotroph colonization or to fix nitrogen or to form nodules with nitrogen-fixing symbiotic rhizobia.

## Introduction

Cereals are grasses from the Poaceae family that were domesticated several thousand years ago in different geographical regions in order to take advantage of the edible components of their grain. Maize, rice, wheat, and sorghum are the most widely grown cereals consumed by humans and this review will focus on these crops. Nitrogen availability often limits cereal crop production. Adding nitrogen to crops has enhanced food production and has consequently increased the human population. In fact, the Haber-Bosch process that produces nitrogen fertilizers industrially has been called the detonator of human population growth (Smil, 2002; Erisman et al., 2008). Increases in food production are urgently needed, yet fertilizers have already been overused, are expensive and polluting. Trends in crop management and genetics predict that crop production will not meet projected food needs in 2050 (Ray et al., 2013). Higher agricultural production will require enormous additional inputs of nitrogen. Cereal production is highly dependent on chemical nitrogen fertilizers and the excessive use of these fertilizers is negatively impacting human and environmental health, including significant effects on the generation of greenhouse gasses and a reduced ozone layer (Reddy et al., 2002; Stokstad, 2016). With the future menaces of a decline in petroleum reserves used in the Haber-Bosch process to produce inorganic fertilizers, besides the low efficiency with which plants use chemical nitrogen fertilizers, researchers are now seriously considering alternate sources of nitrogen for crop production.

Biological nitrogen fixation (BNF) is a potentially attractive alternative source of nitrogen for cereal production (Ladha and Reddy, 1995; Beatty and Good, 2011; Rogers and Oldroyd, 2014). In fact, BNF by diazotrophic bacteria, which reduce dinitrogen to ammonium using nitrogenase enzyme systems, is the major contributor to the nitrogen economy of the biosphere, accounting for 30–50% of the total nitrogen in crop fields (Ormeño-Orrillo et al., 2013). Nitrogen fixation is an energetically expensive process. In theory, nitrogen fixation could fall under the black queen hypothesis (Morris et al., 2012). This hypothesis predicts that in communities of free-living microorganisms, there are only a few “helpers” that have costly functions, such as nitrogen fixation, that support the “beneficiaries” that are dependent on them for nitrogen supplies (Morris et al., 2012). Consequently, diazotrophs generally correspond to minor components of the ecosystems. Diazotrophs are found among alphaproteobacteria, gammaproteobacteria, Firmicutes, betaproteobacteria, and cyanobacteria but do not seem to be the most abundant (dominant) bacteria in plant rhizospheres, so there are possibilities for increasing nitrogen-fixation by favoring their populations. To enhance their competitiveness, plants may be selected or modified to increase exudation of nutrients that would favor the growth of diazotrophs (see below). Additionally, regular inoculation with diazotrophs as is common for legumes, could provide enough bacterial cells for the plant even if bacteria do not persist long in soils. Besides, low soil persistence may not be a disadvantage because it would allow subsequent introductions of more efficient symbionts as inoculants. Inoculant formulations and survival of inoculated bacteria are not within the scope of this review.

Removing plant products from agricultural fields leads to nitrogen and other nutrient deficiencies. Therefore, achieving nitrogen fixation in cereals, like that which occurs in legumes, has been a long-cherished goal and has been considered as a holy “grail” (Triplett, 1996). A huge interest in rice nitrogen fixation is reflected in books devoted to this subject (Khush and Bennett, 1992; Ladha and Reddy, 2000). For many years, researchers have isolated, identified and tested a very large diversity of rhizospheric or endophytic isolates from plants. The practical aim has been to identify nitrogen-fixing bacteria that could be used as crop inoculants, but this has had limited practical success. The experience from efforts to increase nitrogen fixation in legumes showed contrasting results. Hypernodulating soybean plants resulted in diminished yields in some cases (Pracht et al., 1994), but in others there was an increased productivity in subsequent crops (Song et al., 1995).

Diverse microbes are found associated with plants (Bulgarelli et al., 2012, 2013, 2015; Lundberg et al., 2012; Peiffer et al., 2013). There are comprehensive reviews on rhizospheric microbiota (Berg, 2009; Saharan and Nehra, 2011; Mendes et al., 2013), diazotrophs (Santi et al., 2013) and endophytes (residing inside plant tissues, Rosenblueth and Martínez-Romero, 2006; Guo et al., 2008; Liu et al., 2017a) of diverse plants including cereals, all focusing on bacteria. Rhizospheric and endophytic bacteria contribute to plant growth promotion by producing plant hormones, inhibiting pathogens or by enhancing mineral availability (Matiru and Dakora, 2004; Rosenblueth and Martínez-Romero, 2006; Friesen et al., 2011). In most cases, there is not sufficient evidence to consider that nitrogen fixation is a leading cause of plant growth promotion. For example, there are many reports on the growth-promoting effects of *Azospirillum* inoculation in maize, wheat, rice, and sorghum but these will not be reviewed here because the main beneficial effects are not primarily attributed to nitrogen fixation.

In general, the contribution of nitrogen fixation in non-legumes is limited, however, *Beijerinckia* spp. inoculants promoted significant increases in nitrogen content in some maize hybrids (Govedarica, 1990). In contrast to what occurs in nodules, it is common that free-living nitrogen-fixing bacteria (diazotrophs) do not excrete nitrogen compounds to the host plant with ammonium instead being assimilated and used by bacteria for their own growth. The use of genetically modified bacteria was shown to improve plant growth through nitrogen fixation. For example, ammonium excreting *Azospirillum* exhibited enhanced nitrogen supply to wheat plants (Van Dommelen et al., 2009). Similar mutants of *Azospirillum*, *Kosakonia*, *Pseudomonas*, and *Azotobacter* (Zhang et al., 2012; Setten et al., 2013; Geddes et al., 2015; Ambrosio et al., 2017; Bageshwar et al., 2017) proved capable of stimulating plant growth. We would recommend obtaining ammonium-excreting mutants of *Paraburkholderia*, *Herbaspirillum*, or *Azoarcus* as well, to test if they also improve plant growth through nitrogen fixation. Recently, Setten et al. (2013) engineered a root-colonizing non-diazotrophic endophyte, *Pseudomonas protegens* Pf-5, by transferring a stretch of DNA with 52 genes including the *nif* gene cluster from *P. stutzeri* (Vermeiren et al., 1999). The modified *P. protegens* strain fixed nitrogen constitutively, even in the presence of combined nitrogen, and released significant quantities of ammonium into the surrounding medium. In greenhouse tests, Fox et al. (2016) demonstrated increased yields in maize and wheat inoculated with this engineered strain, and ^15^N isotope dilution analysis confirmed that this positive effect was clearly due to nitrogen fixation in roots.

In this review we present additional information about associative nitrogen fixation as well as studies on the genetic modification of cereals directed toward obtaining nitrogen-fixing plants by the transfer of nitrogenase or nodulation genes into plants.

## Sources of Diazotrophic Bacteria

Bacteria can get on to the plants either by root colonization from soil carryover, leaf litter (Pfeiffer et al., 2013), inoculation or via seed transmission. Seed endophytes can migrate from the seed and colonize the plant xylem but can also migrate from beneath the seed coats with the emerging root or even after the seed has germinated (Johnston-Monje and Raizada, 2011) and colonize the rhizoplane and rhizosphere. Johnston-Monje and Raizada (2011) found that only a few endophytes are able to spread from the root vascular tissue into the rhizosphere. The contribution of seed endophytes when colonizing the rhizosphere may be better observed in soils with low bacterial diversity.

Seed-borne pathogens spread and perpetuate bacteria in new plant generations, similarly seeds may also carry beneficial bacteria that may be inherited to new generations. Non-pathogenic seed bacteria have been identified in *Phaseolus vulgaris*, maize, rice, wheat, alfalfa, and other plants (Okunishi et al., 2005; Rijavec et al., 2007; Johnston-Monje and Raizada, 2011; Hardoim et al., 2012; López-López et al., 2012; Liu et al., 2017b; López et al., 2018). Previous analyses of seed endophytes have shown a large diversity of bacteria (Rijavec et al., 2007; Johnston-Monje and Raizada, 2011; López-López et al., 2012; Rosenblueth et al., 2012; Chimwamurombe et al., 2016). Seed isolates from different plants are able to produce auxins, gibberellins, siderophores and ACC deaminase, solubilize phosphates, protect plants against pathogens and fix nitrogen (Zawoznik et al., 2014; Díaz Herrera et al., 2016; Khalaf and Raizada, 2016; Shahzad et al., 2016; Wang et al., 2016; Liu et al., 2017b; Verma et al., 2017).

There are very few studies that analyze bacterial genes required for seed colonization (Molina et al., 2006; Peralta et al., 2016). A *P. putida* mutant in a secretion system had reduced capacity to colonize maize seeds (Molina et al., 2006). Maize rhizospheric bacteria are more numerous and more diverse (Chelius and Triplett, 2001; Gomes et al., 2001; Schmalenberger and Tebbe, 2003; Chauhan et al., 2011; Pereira et al., 2011; Li et al., 2014) than seed endophytes (Rijavec et al., 2007; Johnston-Monje and Raizada, 2011; Rosenblueth et al., 2012), suggesting a bottleneck in the acquisition of bacteria by seeds.

Seeds may also be colonized by bacteria present on the surfaces of stems, flowers, and fruits (Compant et al., 2011; Hardoim et al., 2012; Mitter et al., 2017), as well as from pollen grains, which also harbor bacteria (Madmony et al., 2005; Fürnkranz et al., 2012) that can colonize the ovules after pollination (Agarwal and Sinclair, 1996). Rhizospheric bacteria seem to be mainly acquired from the soil or from leaf litter (Pfeiffer et al., 2013). Dependant on crop management history (Isobe and Ohte, 2014) or soil pH (Andrew et al., 2012; Hardoim et al., 2012). Root endophytic bacteria are acquired from the rhizosphere and a fraction of them can move through the xylem to colonize aerial parts, including seeds (James et al., 2002; Okunishi et al., 2005; Compant et al., 2011; Liu et al., 2017a).

**FIGURE 1:**
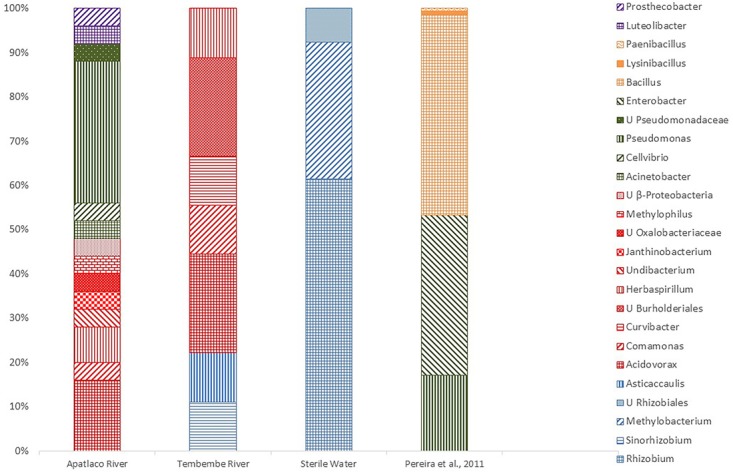
Comparison of bacterial genera composition found in maize rhizospheres. Sequences from 16S rRNA gene were recovered from plantlets watered with Apatlaco or Tembembe rivers or with sterile water. Results from Pereira et al. (2011) obtained by a culture dependent approach are also shown. 16S rRNA sequences that had an identity greater than 95% to other sequences were considered from the same genus. U = unclassified.

To study novel sources of maize associated bacteria, we analyzed the contribution of irrigation and identified bacteria from the maize rhizoplane by sequence analysis of 16S rRNA gene amplicons from plants that were irrigated with water from two different Mexican rivers, Apatlaco and Tembembe (Merino-Flores, 2012). The maize rhizoplane irrigated with river water had river-borne bacteria, previously identified as *Pseudomonas* (Sachman-Ruiz et al., 2009) and there were common bacterial species in maize roots irrigated with water from both rivers, such as *Acidovorax*, *Commamonas*, and *Herbaspirillum* (**Figure 1**). From controls, irrigated with sterile water, only alphaproteobacteria from the Rhizobiales order were observed (**Figure 1**) and identified as *Methylobacterium* and *Rhizobium*. As these bacteria were recovered from plants that were maintained under sterile conditions in sterile vermiculite, irrigated with sterile water and derived from surface-disinfected seeds, they probably derived from kernel endophytes that found their way out of seeds to colonize the rhizosphere. However, seed-borne bacteria were outcompeted in roots by irrigation-borne bacteria, thus lowering the proportion of seed bacteria in the final composition of the plant microbiome from river water irrigated plants. Bacterial genera identified in the maize rhizoplane by a culture-dependent approach (Pereira et al., 2011) were included in this comparison (**Figure 1**). Pereira et al. (2011) found many Firmicutes in the rhizoplane, similar to other reports (Han et al., 2011; Compant et al., 2013). Previously we reported that each kernel had a different subset of endophytes, even when kernels belonged to the same cob (Rosenblueth et al., 2012). This indicated that not all seedlings in a germinating population would have the same bacteria, that would add biodiversity to plants and perhaps bring adaptive advantages.

## Culture-Independent Identification of Diazotrophs in Cereals

By using a culture-independent approach, a better representation of existing diazotrophs may be obtained while the analysis of *nifH* transcripts has provided results on active diazotrophs. This approach based on *nif* gene amplification and sequencing has been used to identify nitrogen-fixing bacteria associated with rice, sorghum, wheat and maize. There are no universal *nif* gene primers and diverse primers should be used to identify different genera or nitrogen-fixing bacterial families.

In rice, a diversity of putative diazotrophs have been revealed by PCR amplification and sequencing of metagenomic DNA from roots. Ueda et al. (1995) reported 23 *nifH* sequences from *Oryza sativa* cv. Nihonn in Japan which grouped with deltaproteobacteria (*Desulfovibrio*), gammaproteobacteria (*Klebsiella*-like, *Azotobacter*) and alphaproteobacteria (*Thiobacillus*-like) genes, the former two groups being the most abundant. Sixteen *nifH* sequences from rice analyzed by Engelhard et al. (2000) from DNA from root macerates clustered with genes from alphaproteobacteria (*Bradyrhizobium*, *Azorhizobium*, and *Rhizobium*) and betaproteobacteria (*Azoarcus*) as well with those from Firmicutes (related to *Clostridium*). Proteobacteria was also dominant among *nifH* sequences obtained from DNA extracted from rice in Thailand with betaproteobacteria being the most abundant (e.g., *Herbaspirillum*) (Rangjaroen et al., 2014).

Diazotrophs expressing nitrogenase reductase mRNA in association with rice have been identified. Elbeltagy and Ando (2008) sequenced 117 *nifH* transcripts from *O. sativa* cv. Sprice and cv. Tetep grown in Japan and found that most sequences (>70%) belonged to a novel cluster related to *Geobacter sulfurreducens* (deltaproteobacteria), other sequences were affiliated with alphaproteobacteria (*Bradyrhizobium* and *Methylocystis*), betaproteobacteria (*Azovibrio*), gammaproteobacteria (*Azotobacter*), Firmicutes (related to *Heliobacterium*) and a polyphyletic group encompassing anaerobes. *Azoarcus* seemed as a dominant active nitrogen fixer in mixed rhizosphere/root samples from rice cultivated in a paddy soil in China (Wartiainen et al., 2008) while Mårtensson et al. (2009) found an abundance of proteobacteria-related sequences from the alpha, beta and gamma subdivisions as well as fewer sequences from a polyphyletic anaerobe group when studying samples from the same site just one year later. In a proteomic approach, dinitrogen reductase proteins from *Bradyrhizobium*, *Magnetospirillum*, and *Azospirillum* have been detected in the rhizosphere of rice growing in Philippines (Knief et al., 2012).

Among 245 *nifH* sequences obtained from soil DNA of sorghum (*Sorghum bicolor*) rhizospheres cultivated in Brazil, Coelho et al. (2008) found mostly proteobacterial diazotrophs. Sequences were related to bacteria from the Rhizobiales, Burkholderiales, Sphingomonadales, Rhodospirillales, Enterobacteriales, and Bacillales. Diazotrophs with *nifH* sequences >98% identical to those of *Bradyrhizobium* and *Rhizobium* were common.

The identity of diazotrophs inhabiting the rhizosphere and different tissues of maize (roots and stems) was determined by Roesch et al. (2008) by sequencing *nifH* amplified from DNA extracts. They found that Proteobacterial sequences were the most prevalent in all analyzed tissues and also in the rhizosphere. Members of the alpha, beta, gamma, or delta subdivisions were present but the two former subdivisions were numerically dominant. The most abundant genera were *Azospirillum*, *Bradyrhizobium*, *Herbaspirillum, Ideonella*, *Klebsiella*, and *Raoultella*.

Naturally-occurring diazotrophs were analyzed in all previous studies but some studies have evaluated the expression of nitrogenase genes of inoculated strains. You et al. (2005) reported the expression of *nifH* of *Herbaspirillum* sp. B501 in shoots of wild rice (*O. officinalis*) after inoculation of the bacteria to the seeds. *Azospirillum brasilense* FP2 applied to wheat (*Triticum aestivum*) was shown to express the *nifHDK* operon when colonizing roots (Camilios-Neto et al., 2014). Another inoculant strain, *Herbaspirillum seropedicae* SmR1, was also able to express the same genes when attached to wheat roots (Pankievicz et al., 2016).

From these studies, it can be concluded that a wide diversity of bacteria possessing *nifH* genes are associated with cereals. It is worth noting that a significant proportion of the sequences obtained form clusters that are unrelated to known taxonomic groups. Functional molecular analyses in rice have shown that not all of these microorganisms are active at nitrogen fixation in association with the plant. The taxonomic composition of the active diazotrophs varies from plant to plant but Proteobacteria are always present and a polyphyletic group of anaerobes are very common. The presence of the latter group may be related to the nature of rice cultivation under water. Alphaproteobacteria from the Rhizobiales order, specifically of the *Bradyrhizobium* genus, have been found as active nitrogen fixers both by transcriptomic and proteomic approaches. Interestingly, in sugarcane, another member of the Poaceae family, bradyrhizobia also express nitrogen fixation genes *in planta* (Thaweenut et al., 2011; Fischer et al., 2012). Rhizobia in general have been used as biofertilizers in agriculture for more than a century and it will be worth exploring if bradyrhizobial inoculants can be developed for cereals.

## Diazotrophs May Be Human Pathogens

It is not uncommon to isolate human or plant pathogens from plants and in many cases they are efficient growth promoting bacteria. Their use in agriculture should not be encouraged. Among the nitrogen-fixing bacteria isolated from cereals there are human pathogens or potential human pathogens (Berg et al., 2005), such as *Burkholderia cepacia* and *Klebsiella variicola* (Rosenblueth et al., 2004, 2011; Kutter et al., 2006; Martínez-Romero et al., 2018). *B. cepacia* complex (BCC) includes seventeen species, some of them responsible for potentially lethal pulmonary infection in immuno-compromised or cystic fibrosis patients, and others are causative agents of infection in animals and plants (Sawana et al., 2014). Members of this complex are generally good colonizers of plant rhizosphere and possess traits to improve plant growth (Fiore et al., 2001; Mendes et al., 2007). For a long time, *B. vietnamensis*, found in the rhizosphere and rhizoplane of maize, coffee, and sorghum plants (Gillis et al., 1995) was recognized as the only nitrogen-fixing species belonging to the BCC. However, new species of plant-associated *Paraburkholderia* diazotrophs have been reported, such as *P. unamae*, *P. tropica*, *P. xenovorans* from rhizospheric or endophytic association with maize, coffee, sorghum, or sugarcane (Perin et al., 2006). This group of bacteria has been found to comprise mainly environmental and plant-associated isolates (Baldani et al., 2000; Chen et al., 2003; Govindarajan et al., 2006). The presence of two transmissible virulence factor genes such as the *cblA* (encoding giant cable pili) and the epidemic strain marker regulator (*esmR)* identified among clinical isolates of opportunistic pathogens of *B. cenocepacia* and other species of the BCC have not been detected by PCR amplification and ^32^P hybridization in the environmental diazotrophic isolates of *B. unamae* and *B. tropica*. Thus, the lack of the aforementioned pathogenic traits supports the potential for using them as plant growth-promoting bacteria, since they were shown to have the ability to improve maize growth (Perin et al., 2006). Genomic analysis of the plant-associated *Burkholderia* and the pathogen *B. cenocepacia* for the occurrence of virulence determinants implicated in pathogenesis as well as the functional tests to determine pathogenicity showed that these two phylogenetic groups of *Burkholderia* belong to two distinct lineages. Mutualistic strains did not possess the virulence determinants tested and were susceptible to the vast majority of antibiotics. They did not kill *Caenorhabditis elegans* nor lyse of HeLa cells, unlike the pathogen *B. thailandensis* (Angus et al., 2014). Abundance of phylogenetic, biochemical, and molecular evidences for the occurrence of two different lineages within the genus *Burkholderia* finally led to a taxonomic revision with a split in the genus and allowed the environmental group to be renamed as *Paraburkholderia*, which nevertheless still includes a few human clinical isolates (Estrada-de los Santos et al., 2013; Angus et al., 2014; Sawana et al., 2014). Thus, some of the isolates that were formerly classified as *Burkholderia* species may still be considered suitable candidates as plant growth promoters.

*Klebsiella* has been isolated from several distinct plants (Chelius and Triplett, 2000; Martínez et al., 2003; Rosenblueth et al., 2004; Martínez-Romero et al., 2015; Liu et al., 2017b; Reyna-Flores et al., 2018). However, its use in agriculture has been discouraged since some strains of *Klebsiella* were found to be pathogens or opportunistic pathogens of humans and animals (Martínez et al., 2004; Davidson et al., 2015; Martínez-Romero et al., 2018). Comparative genomic analysis of *K. variicola* 342 (originally reported as *K. pneumoniae*) and *K. pneumoniae* MGH78578 showed that the latter cannot fix nitrogen, and there was a difference between these two species in the presence of genes essential for attachment, transport, and secretion. *K. variicola* 342 possesses genes that are involved in processing plant-derived cellulose and aromatic compounds but did not have a Type III secretion system that can be used to secrete effector proteins into the cytoplasm of eukaryotic cells, promoting their infection, nor genes encoding effector proteins. It was resistant to antibiotics (Fouts et al., 2008). However, in the experiments conducted in mice to test pathogenicity, *K. variicola* 342 caused urinary tract infection like the clinical isolate C3091 but showed a significantly lower level of lung infection.

On the other hand, other plant-associated bacteria such as *Azospirillum* (Okon and Itzigsohn, 1995), *Herbaspirillum* (Baldani et al., 2000), *Gluconacetobacter diazotrophicus* (Muthukumarasamy et al., 2005) and *Azoarcus* (Reinhold-Hurek and Hurek, 1997) are considered safe and they are used as inoculants in agriculture. *Azoarcus* and *Herbaspirillum* have been shown to fix nitrogen in rice (Elbeltagy et al., 2001; Hurek et al., 2002; Roncato-Maccari et al., 2003). The complete genome sequence of *Herbaspirillum seropedicae* SmR1, a spontaneous streptomycin resistant mutant, reveals it to be a metabolically versatile bacterium that contains genes coding for degradation of aromatic compounds. The limited number of genes related to mobile elements suggests a low rate of DNA transfer in this microorganism that is presumably due to adaptation to a stable microenvironment. *H. seropedicae* can synthesize plant-growth promoting substances such as auxins and gibberellins, and fixes nitrogen. It has a variety of protein secretion systems involved in plant bacterial recognition (Pedrosa et al., 2011). Likewise, *Azoarcus* sp. BH72 genome contains few mobile elements in comparison to many soil bacteria or pathogens, which indicates that its genome has low plasticity. The strain appears to be “disarmed” compared to plant pathogens due to lack of known toxins as well as Types III and IV secretion systems. The lack of a *N*-acyl homoserine lactone-based communication system argues for a rather exclusive microhabitat and, the presence of genes coding for nitrogen fixation, chemotaxis, iron acquisition and biocontrol offer insight into genomic strategies for an endophytic life style and allow identification of various features that contribute to its interaction with plants (Krause et al., 2007).

Rhizobia are also considered safe and have been used in agriculture as legume inoculants for more than one hundred years and their use in non-legumes is recommended as well. Bradyrhizobia have been found to be associated with wild rice in Africa (Chaintreuil et al., 2000), sweet potatoes in Japan (Terakado-Tonooka et al., 2008) and sugarcane in Brazil (Fischer et al., 2012). Some *Rhizobium* genotypes are very efficient at colonizing maize roots (Gutiérrez-Zamora and Martínez-Romero, 2001; Rosenblueth and Martínez-Romero, 2004). Rhizobia promote rice growth (Yanni et al., 1997) perhaps due to nitrogen fixation, and we suppose that bradyrhizobia may contribute fixed nitrogen to plants since some bradyrhizobial strains are capable of fixing nitrogen in the free-living state. A general brief overview of the rhizobial genetic repertoire to colonize non-legumes was published (López-Guerrero et al., 2013).

## Plants as Determinants of Bacterial Interactions

Nitrogen fixation is highly variable depending on the associated diazotroph and the plant variety, but the host plant exerts a determinant effect by supplying the carbon and energy source for bacterial growth and nitrogen fixation. Aluminum or acid tolerant plants were found to sustain high levels of nitrogen fixation due to the exudation of dicarboxylic acids from their roots (Christiansen-Weniger et al., 1992). The maize rhizosphere is a habitat favorable for diazotroph proliferation due to high quantities of exudates [accounting for 20–40% of all photosynthate (Stevenson and Cole, 1999)], although unbalanced in C and N. Root exudates and plant secondary metabolites have a selective or inhibitory effect on bacteria (Guntli et al., 1999; Bending and Lincoln, 2000). Sphingomonadales prefer root exudates from monocotyledonous plants rather than from other plants (Haichar et al., 2008), but it may be premature to make general statements. Plant species, genotype, and age have effects on root microbiota (Dalmastri et al., 1999; Cavaglieri et al., 2009; Hartmann et al., 2009; Peiffer et al., 2013; Chaparro et al., 2014; Johnston-Monje et al., 2014; Wagner et al., 2016; Pfeiffer et al., 2017).

Phytoalexins and salicylic acid that mediate plant defense in legumes have inhibitory effects on plant-*Rhizobium* interactions (Parniske et al., 1991; González-Pasayo and Martínez-Romero, 2000; Stacey et al., 2006; Lebeis et al., 2015), but less is known about the effects of defense alkaloids on diazotrophs in cereals. Maize bacillus and rhizobial endophytes were found to be resistant to MBOA (Rosenblueth and Martínez-Romero, 2004), which is a toxic allelochemical in maize (Abel et al., 1995). Salicylic acid from plants restricts bacterial root colonization (Lebeis et al., 2015). Additionally, plants may interfere or stimulate quorum sensing signaling among bacteria (Bauer and Mathesius, 2004; Venturi and Keel, 2016), which may have significant effects by changing bacterial gene expression.

## Prospects for Engineering Cereal Plants

Besides using associated bacteria to provide nitrogen to cereals other strategies involving the genetic modification of plants have been considered and are reviewed here. Two major approaches, transferring nitrogenase genes into crop plants and the development of the root nodular symbiosis in cereals, were envisioned as important avenues for achieving this target (Ladha and Reddy, 1995, Ladha and Reddy, 2000; Beatty and Good, 2011). Indeed, all these options have been considered and initial feasibility studies were conducted under the auspices of the International Rice Research Institute-coordinated multinational frontier project on “Assessing Opportunities for Nitrogen Fixation in Rice” during 1994–2001 (see Ladha and Reddy, 2000). However, major breakthroughs in the genomics of diazotrophs and the genetics of nitrogen fixation, as well as the processes involved in legume-rhizobia symbioses in recent years have opened up new avenues to tackle this problem much more systematically and have prompted the formulation of more workable schemes aimed at achieving this goal (Beatty and Good, 2011).

For the goal of generating nitrogen-fixing cereal crops, several analogous projects funded by the Bill and Melinda Gates Foundation (BMGF, United States), the National Science Foundation (NSF, United States), the Biotechnology and Biological Sciences Research Council (BBSRC, United Kingdom) and the Indian Council of Agricultural Research (ICAR, India) have recently been initiated with differential emphasis on the choice of crop or experimental system. Among these, the first approach considers assembling of an active nitrogenase in plants through the incorporation and expression of bacterial genetic machinery to encode and support functional nitrogenase system. Nitrogen fixation is a highly energy demanding process, and so chloroplasts and mitochondria are envisaged as suitable sites for nitrogen fixation since they can meet the energy requirements for nitrogenase in plant cells. Nitrogenase is extremely sensitive to oxygen and irreversibly inactivated in air, and so the oxygen evolved by chloroplasts during photosynthesis may be detrimental to the maintenance of nitrogenase enzyme complex integrity. Thus, expressing functional nitrogenase in chloroplasts requires temporal (day/night) separation of photosynthesis and nitrogen fixation by confining *nif* gene expression only to dark periods (nights) or, alternatively, by spatially restricting *nif* gene expression to non-photosynthetic tissues such as the root system. As a proof of concept using yeast (a non-photosynthetic organism) as a model system, López-Torrejón et al. (2016) engineered *nifH, nifM, nifS*, and *nifU* from *Azotobacter vinelandii* into this eukaryotic cell and showed that active nitrogenase Fe protein can be produced if NifH polypeptide is targeted to the mitochondrial milieu jointly with the NifM maturase. They further demonstrated that for the generation of an active Fe protein, concomitant transfer of the NifH-specific Fe–S cofactor synthesizing protein components NifU and NifS into mitochondria is not essential, because NifH is able to acquire/incorporate endogenously generated mitochondrial Fe–S clusters. In a subsequent study, Buren et al. (2017) targeted a minimum set of nine *A. vinelandii nif* genes (*nifH, nifD, nifK, nifU, nifS, nifM, nifB, nifE*, and *nifN*) into mitochondria and demonstrated successful formation of NifDK tetramer, an essential first step in assembling a functional nitrogenase in a eukaryotic cell. *nif* gene transfer has also been attempted in plants. Ivleva et al. (2016) expressed NifH protein together with nifM in chloroplasts of tobacco plants, generating functional NifH, although with low activity. Recently, Allen et al. (2017) demonstrated the feasibility of expressing the complete range of biosynthetic and catalytic nitrogenase (Nif) proteins as mitochondrial targeting transit peptide-Nif fusions in tobacco leaves. Studies in both yeast and tobacco showed, however, that NifD polypeptide is prone to degradation in eukaryotic cells (Allen et al., 2017; Buren et al., 2017), thus warranting a need for optimizing its amino acid sequence to improve stability without compromising catalytic activity. We suggest that readers refer to the excellent recently published review articles for a comprehensive account of the strategy for *nif* gene transfer to eukaryotes (Curatti and Rubio, 2014; Buren and Rubio, 2017).

The second approach envisions the development of legume-like root-nodule symbioses (RNS) in cereal crop plants (Reddy et al., 2013; Rogers and Oldroyd, 2014). This approach is based on contemporary knowledge on the development of the endosymbiotic associations of most land plants with endomycorrhizal fungi that form phosphate-acquiring arbuscular mycorrhizae (AM) in cereals and legumes, and with diverse diazotrophic rhizobia, to form nitrogen-fixing RNS in legumes. Genetic constituents that are critical for triggering initial processes for the development of AM symbiosis (AMS) are similar in both legumes and rice, and possibly in other cereals too (Gutjahr et al., 2008). Moreover, in legumes, these same genetic components play a critical role in aiding initial stages of RNS development as well. Thus, genetic elements that participate in promoting both AMS and RNS development constitute the “common symbiosis pathway” (CSP; Markmann and Parniske, 2008). Current lines of research in cereals are making use of functionally conserved genetic constituents of the CSP as a foundation to extend genetic networks to assemble a complete signaling pathway to support legume-like RNS in cereal crops (Reddy et al., 2013; Rogers and Oldroyd, 2014; Delaux et al., 2015; Mus et al., 2016).

An alternative option is to develop cereals that promote the growth of diazotrophs. Since the population density of endophytic bacteria in plant tissues is too low to support adequate nitrogen fixation, it is important to design systems that aid greater colonization of diazotrophic endophytes for improved nitrogen fixation in the crop plants. To achieve this, it is critical to improve the chances that the inoculated diazotroph will selectively colonize the crop plant. This is essential because newly introduced bacterial strains are usually out-competed by the native microbial communities in the rhizosphere of plants. This impediment could be surmounted by engineering plants to produce a specific metabolite and thus create a “biased rhizosphere” to favor the growth of an introduced diazotroph able to use the novel metabolite (Rossbach et al., 1994).

## Would Carbon Costs Incurred Due to *In Planta* Nitrogen Fixation Reduce Crop Yields in Cereals?

Nitrogen fixation is a highly energy requiring process and the factors that limit symbiotic nitrogen fixation have been analyzed in only few legumes. For example, oxygen diffusion was found to limit carbon metabolism and nitrogen fixation in nodules (Vance and Heichel, 1991). In legumes such as soybean, energy costs are significant for both N_2_ fixation and NO_3_ assimilation but are apparently somewhat greater for the former. Layzell (2000) estimated that in soybean, 5 CO_2_ are released per N_2_ fixed, while during nitrate assimilation approximately 5.7 CO_2_ are released per nitrogen assimilated in non-photosynthetic tissue, and 0–2.9 CO_2_ per nitrogen assimilated in photosynthetic tissue (see Ladha and Reddy, 2000 for detailed discussion). While there is no doubt that supplying ammonia (NH_3_) as a nitrogen source for plants reduces the energy requirement for nitrogen assimilation, the proper comparison that should most often be made (except for paddy rice) is between dinitrogen (N_2_) and nitrate as nitrogen sources, since nitrate is the most common alternative nitrogen source available in aerated soils. In plants that use nitrate as a source of nitrogen, nitrate first needs to be converted to ammonia to allow the synthesis of amino acids. Nitrate uptake and its conversion into ammonia is an energy requiring process. It has been estimated that carbon or energy costs for the conversion of NO_3_ to NH_4_^+^ is: ΔG = -605 kJ mol^-1^ (Pate et al., 1979; Kennedy and Cocking, 1997). Likewise, in the case of nitrogen-fixing legumes, N_2_ is first converted into NH_4_^+^, and then into amino acids. It is calculated that carbon or energy costs for the conversion of N_2_ to NH_4_^+^ is about -687 kJ mol^-1^. These theoretical carbon or energy costs for conversion of NO_3_ and N_2_ to NH_4_^+^ are quite similar.

In legumes, there is no experimental evidence to support the contention that nitrogen fixation reduces yield. Fertilization in field conditions with various forms of combined nitrogen rarely produced any significant advantage to final yield of plants (Vance and Heichel, 1991). Under greenhouse conditions as well, no significant yield differences were observed when the plants were grown on dinitrogen versus nitrate as a nitrogen source (Gibson, 1966). This indicates that legumes using BNF rather than nitrate nitrogen suffer no obvious yield penalties. Urea and ammonium sulfate are normally used to fertilize rice. An important point to note here is that in spite of the greater energy requirement for nitrate assimilation (compared to ammonia assimilation), rice yields are better when grown on nitrate combined with ammonia as compared to ammonia alone (Xiaoe and Xi, 1991; Ancheng et al., 1993). The fact that no yield penalty exists for rice grown on nitrate and ammonia rather than ammonia alone suggests that energy may not be limiting.

The ability of plants to compensate for extra energy consumption cannot be ignored, as photosynthetic systems saturate at relatively low light intensity. Nevertheless, since source and sink metabolisms are tightly coupled, it is reasonable to assume that the extra energy consumption by roots would stimulate the production of biomass in shoots. It is well established that equilibrium between photosynthetic sugar synthesis in the chloroplast-containing leaf cells (source tissues) and sugar consumption by roots, fruits and grains (sink tissues) must be maintained for sustaining plant growth and survival. In plants under optimal light and at the normal carbon dioxide levels, sink limitation occurs when the rate of photosynthesis is limited by insufficient withdrawal of photosynthetic products generated in the green tissues through the Calvin–Benson cycle (Sawada et al., 1986; Sharkey et al., 1986; Paul and Foyer, 2001; Adams et al., 2013).

It is intuitively envisaged that a nitrogen-fixing symbiosis in rice may be such a strong sink for photosynthate that yields would be impacted. Since rice is low in protein, a much lower rate of nitrogen fixation than in protein-rich legumes will be needed, with less demand for the plant’s photosynthates (Ladha and Reddy, 1995). In cereals, it has been estimated that as much as 29% of photosynthate is released as exudates by roots into the rhizosphere (Lynch and Whipps, 1990). From this, it may be inferred that cereals like rice have a capacity to sustain carbon/energy costs to support nitrogen fixation (through utilization of root exudates) without causing any strain on their productivity. Also, incidentally, in rice the actual grain yields are considerably lower than their maximum genetic potential. Therefore, *in planta* nitrogen-fixing attribute may not significantly impact the present yield levels (Ladha and Reddy, 2000).

## Conclusion and Perspectives

There has been a biotechnological interest to promote associative nitrogen fixation in non-legume crops that normally use large amounts of chemical fertilizers. Different nitrogen-fixing bacteria have been isolated from cereal roots by culture-dependent methods, and when used as plant inoculants they have varying degrees and strategies for plant growth promotion (Kennedy et al., 2004; Bhattacharjee et al., 2008; Santi et al., 2013). Some past efforts to increase nitrogen fixation in cereals by promoting pseudonodules with phytohormones failed. Notably, recently obtained ammonium excreting mutants of some plant-associated diazotrophs were effective for promoting plant growth suggesting that they became capable of supplying nitrogen to their hosts. Even though achieving genetically-modified nitrogen-fixing cereal crops is a complex process, the approaches that are being pursued at present are creating exciting possibilities for generating such plants in the foreseeable future. If so, the global environmental benefits of a reduced chemical fertilizer usage will be large, and we suppose that detrimental ecological consequences of nitrogen fixing cereals will be minimal. Besides nitrogen, other agricultural inputs, such as phosphorus and water, may limit crop productivity. Mycorrhiza and plant cultivars with high phosphate use efficiency should be considered when developing nitrogen fixing cereals. However, we consider that not only the use of microbes and genetically modified plants will be required to achieve this goal, but a better crop management and efficient programs to control human population-growth are needed as well.

## Author Contributions

MR wrote part of the “Introduction” and “Sources of Diazotrophic Bacteria,” contributed to the discussion and prepared the figure. EO-O wrote the part “Culture-Independent Identification of Diazotrophs in Cereals” and contributed to the discussion. AL-L wrote the part “Diazotrophs May Be Human Pathogens.” BR-H and MR contributed to the discussion and performed the laboratory experiments. JM-R contributed to the discussion, searched for references, and corrected the manuscript. PR wrote the parts “Prospects for Engineering Cereal Plants” and “Would Carbon Costs Incurred Due to *in planta* Nitrogen Fixation Reduce Crop Yields in Cereals?” EM-R wrote the parts “Abstract,” “Conclusion and Perspectives,” and “Plants as Determinants of Bacterial Interactions,” and also assembled the paper and coordinated the study.

## Conflict of Interest Statement

The authors declare that the research was conducted in the absence of any commercial or financial relationships that could be construed as a potential conflict of interest.

## References

[B1] Abel C. A. Wilson R. L. Robbins J. C. (1995). Evaluation of Peruvian maize for resistance to European corn borer (Lepidoptera: Pyralidae) leaf feeding and ovipositional preference. *J. Econ. Entomol.* 88 1044–1048. 10.1093/jee/88.4.1044

[B2] Adams W. W. III Muller O. Cohu C. M. Demmig-Adams B. (2013). May photoinhibition be a consequence, rather than a cause, of limited plant productivity? *Photosynth. Res.* 117 31–44. 10.1007/s11120-013-9849-7 23695654

[B3] Agarwal V. K. Sinclair J. B. (1996). *Principles of Seed Pathology.* Boca Raton, FL: Lewis Publication.

[B4] Allen R. S. Tilbrook K. Warden A. C. Campbell P. C. Rolland V. Singh S. P. (2017). Expression of 16 nitrogenase proteins within the plant mitochondrial matrix. *Front. Plant Sci.* 8:287. 10.3389/fpls.2017.00287 28316608 PMC5334340

[B5] Ambrosio R. Ortiz-Marquez J. C. Curatti L. (2017). Metabolic engineering of a diazotrophic bacterium improves ammonium release and biofertilization of plants and microalgae. *Metab. Eng.* 40 59–68. 10.1016/j.ymben.2017.01.002 28089747

[B6] Ancheng L. Jianming X. Xiaoe Y. (1993). Effect of nitrogen (NH_4_NO_3_) supply on absorption of ammonium and nitrate by conventional and hybrid rice during reproductive growth. *Plant Soil* 15 395–398. 10.1007/BF00025066

[B7] Andrew D. R. Fitak R. R. Munguia-Vega A. Racolta A. Martinson V. G. Dontsova K. (2012). Abiotic factors shape microbial diversity in Sonoran Desert soils. *Appl. Environ. Microbiol.* 78 7527–7537. 10.1128/AEM.01459-12 22885757 PMC3485727

[B8] Angus A. Agapakis C. M. Fong S. Yerrapragada S. Estrada-de los Santos P. Yang P. (2014). Plant-associated symbiotic *Burkholderia* species lack hallmark strategies required in mammalian pathogenesis. *PLoS One* 9:e83779. 10.1371/journal.pone.0083779 24416172 PMC3885511

[B9] Bageshwar U. K. Srivastava M. Pardha-Saradhi P. Paul S. Gothandapani S. Jaat R. S. (2017). An environment friendly engineered *Azotobacter* can replace substantial amount of urea fertilizer and yet sustain same wheat yield. *Appl. Environ. Microbiol.* 83:e00590-17. 10.1128/AEM.00590-17 28550063 PMC5514683

[B10] Baldani V. L. D. Baldani J. I. Döbereiner J. (2000). Inoculation of rice plants with the endophytic diazotrophs *Herbaspirillum seropedicae* and *Burkholderia* spp. *Biol. Fertil. Soils* 30 485–491. 10.1007/s003740050027

[B11] Bauer W. D. Mathesius U. (2004). Plant responses to bacterial quorum sensing signals. *Curr. Opin. Plant Biol.* 7 429–433. 10.1016/j.pbi.2004.05.008 15231266

[B12] Beatty P. H. Good A. G. (2011). Future prospects for cereals that fix nitrogen. *Science* 333 416–417. 10.1126/science.1209467 21778391

[B13] Bending G. D. Lincoln S. (2000). Inhibition of soil nitrifying bacteria communities and their activities by glucosinolate hydrolysis products. *Soil Biol. Biochem.* 32 1261–1269. 10.1016/S0038-0717(00)00043-2

[B14] Berg G. (2009). Plant-microbe interactions promoting plant growth and health: perspectives for controlled use of microorganisms in agriculture. *Appl. Microbiol. Biotechnol.* 84 11–18. 10.1007/s00253-009-2092-7 19568745

[B15] Berg G. Eberl L. Hartmann A. (2005). The rhizosphere as a reservoir for opportunistic human pathogenic bacteria. *Environ. Microbiol.* 7 1673–1685. 10.1111/j.1462-2920.2005.00891.x 16232283

[B16] Bhattacharjee R. B. Singh A. Mukhopadhyay S. N. (2008). Use of nitrogen-fixing bacteria as biofertiliser for non-legumes: prospects and challenges. *Appl. Microbiol. Biotechnol.* 80 199–209. 10.1007/s00253-008-1567-2 18600321

[B17] Bulgarelli D. Garrido-Oter R. Münch P. C. Weiman A. Dröge J. Pan Y. (2015). Structure and function of the bacterial root microbiota in wild and domesticated barley. *Cell Host Microbe* 17 392–403. 10.1016/j.chom.2015.01.011 25732064 PMC4362959

[B18] Bulgarelli D. Rott M. Schlaeppi K. Ver Loren van Themaat E. Ahmadinejad N. Assenza F. (2012). Revealing structure and assembly cues for *Arabidopsis* root-inhabiting bacterial microbiota. *Nature* 488 91–95. 10.1038/nature11336 22859207

[B19] Bulgarelli D. Schlaeppi K. Spaepen S. Ver Loren van Themaat E. Schulze-Lefert P. (2013). Structure and functions of the bacterial microbiota of plants. *Annu. Rev. Plant Biol.* 64 807–838. 10.1146/annurev-arplant-050312-120106 23373698

[B20] Buren S. Rubio L. M. (2017). State of the art in eukaryotic nitrogenase engineering. *FEMS Microbiol. Lett.* 365:fnx274. 10.1093/femsle/fnx274 29240940 PMC5812491

[B21] Buren S. Young E. M. Sweeny E. A. López-Torrejón G. Veldhuizen M. Voigt C. A. (2017). Formation of nitrogenase NifDK tetramers in the mitochondria of *Saccharomyces cerevisiae*. *ACS Synth. Biol.* 6 1043–1055. 10.1021/acssynbio.6b00371 28221768 PMC5477005

[B22] Camilios-Neto D. Bonato P. Wassem R. Tadra-Sfeir M. Z. Brusamarello-Santos L. C. Valdameri G. (2014). Dual RNA-seq transcriptional analysis of wheat roots colonized by *Azospirillum brasilense* reveals up-regulation of nutrient acquisition and cell cycle genes. *BMC Genomics* 15:378. 10.1186/1471-2164-15-378 24886190 PMC4042000

[B23] Cavaglieri L. Orlando J. Etcheverry M. (2009). Rhizosphere microbial community structure at different maize plant growth stages and root locations. *Microbiol. Res.* 164 391–399. 10.1016/j.micres.2007.03.006 17524636

[B24] Chaintreuil C. Giraud E. Prin Y. Lorquin J. Bâ A. Gillis M. (2000). Photosynthetic bradyrhizobia are natural endophytes of the African wild rice *Oryza breviligulata*. *Appl. Environ. Microbiol.* 66 5437–5447. 10.1128/AEM.66.12.5437-5447.2000 11097925 PMC92479

[B25] Chaparro J. M. Badri D. V. Vivanco J. M. (2014). Rhizosphere microbiome assemblage is affected by plant development. *ISME J.* 8 790–803. 10.1038/ismej.2013.196 24196324 PMC3960538

[B26] Chauhan P. S. Chaudhry V. Mishra S. Nautiyal C. S. (2011). Uncultured bacterial diversity in tropical maize (*Zea mays* L.) rhizosphere. *J. Basic Microbiol.* 51 15–32. 10.1002/jobm.201000171 21259285

[B27] Chelius M. K. Triplett E. W. (2000). Immunolocalization of dinitrogenase reductase produced by *Klebsiella pneumoniae* in association with *Zea mays* L. *Appl. Environ. Microbiol.* 66 783–787. 10.1128/AEM.66.2.783-787.2000 10653751 PMC91896

[B28] Chelius M. K. Triplett E. W. (2001). The diversity of archaea and bacteria in association with the roots of *Zea mays* L. *Microb. Ecol.* 41 252–263. 10.1007/s002480000087 11391463

[B29] Chen W. M. Moulin L. Bontemps C. Vandamme P. Béna G. Boivin-Masson C. (2003). Legume symbiotic nitrogen fixation by β-proteobacteria is widespread in nature. *J. Bacteriol.* 185 7266–7272. 10.1128/JB.185.24.7266-7272.2003 14645288 PMC296247

[B30] Chimwamurombe P. M. Grönemeyer J. L. Reinhold-Hurek B. (2016). Isolation and characterization of culturable seed-associated bacterial endophytes from gnotobiotically grown Marama bean seedlings. *FEMS Microbiol. Ecol.* 92:fiw083. 10.1093/femsec/fiw083 27118727

[B31] Christiansen-Weniger C. Groneman A. F. van Veen J. A. (1992). Associative N_2_ fixation and root exudation of organic acids from wheat cultivars of different aluminium tolerance. *Plant Soil* 139 167–174. 10.1007/BF00009307

[B32] Coelho M. R. de Vos M. Carneiro N. P. Marriel I. E. Paiva E. Seldin L. (2008). Diversity of nifH gene pools in the rhizosphere of two cultivars of sorghum (Sorghum bicolor) treated with contrasting levels of nitrogen fertilizer. *FEMS Microbiol. Lett.* 279 15–22. 10.1111/j.1574-6968.2007.00975.x 18070072

[B33] Compant S. Gangl H. Sessitsch A. (2013). “Visualization of niches of colonization of firmicutes with *Bacillus* spp. in the rhizosphere, rhizoplane, and endorhiza of grapevine plants at flowering stage of development by FISH microscopy,” in *Molecular Microbial Ecology of the Rhizosphere*, Vol. 1-2 ed. Bruijn F. J. de (Hoboken, NJ: John Wiley & Sons, Inc), 10.1002/9781118297674.ch39

[B34] Compant S. Mitter B. Colli-Mull J. G. Gangl H. Sessitsch A. (2011). Endophytes of grapevine flowers, berries, and seeds: identification of cultivable bacteria, comparison with other plant parts, and visualization of niches of colonization. *Microb. Ecol.* 62 188–197. 10.1007/s00248-011-9883-y 21625971

[B35] Curatti L. Rubio L. M. (2014). Challenges to develop nitrogen-fixing cereals by direct *nif*-gene transfer. *Plant Sci* 225 130–37. 10.1016/j.plantsci.2014.06.003 25017168

[B36] Dalmastri C. Chiarini L. Cantale C. Bevivino A. Tabacchioni S. (1999). Soil type and maize cultivar affect the genetic diversity of maize root–associated *Burkholderia cepacia* populations. *Microb. Ecol.* 38 273–284. 10.1007/s002489900177 10541789

[B37] Davidson F. W. Whitney H. G. Tahlan K. (2015). Genome sequences of *Klebsiella variicola* isolates from dairy animals with bovine mastitis from Newfoundland, Canada. *Genome Announc.* 3:e00938-15. 10.1128/genomeA.00938-15 26358587 PMC4566169

[B38] Delaux P. M. Radhakrishnan G. Oldroyd G. (2015). Tracing the evolutionary path to nitrogen-fixing crops. *Curr. Opin. Plant Biol.* 26 95–99. 10.1016/j.pbi.2015.06.003 26123396

[B39] Díaz Herrera S. Grossi C. Zawoznik M. Groppa M. D. (2016). Wheat seeds promoters harbour bacterial endophytes with potential as plant growth of biocontrol agents *Fusarium graminearum*. *Microbiol. Res.* 18 37–43. 10.1016/j.micres.2016.03.002 27242141

[B40] Elbeltagy A. Ando Y. (2008). Expression of nitrogenase gene (*nifH*) in roots and stems of rice, *Oryza sativa*, by endophytic nitrogen-fixing communities. *Afr. J. Biotechnol.* 7 1950–1957. 10.5897/AJB2008.000-5042

[B41] Elbeltagy A. Nishioka K. Sato T. Suzuki H. Ye B. Hamada T. (2001). Endophytic colonization and in planta nitrogen fixation by a *Herbaspirillum* sp. isolated from wild rice species. *Appl. Environ. Microbiol.* 67 5285–5293. 10.1128/AEM.67.11.5285-5293.2001 11679357 PMC93302

[B42] Engelhard M. Hurek T. Reinhold-Hurek B. (2000). Preferential occurrence of diazotrophic endophytes, *Azoarcus* spp., in wild rice species and land races of *Oryza sativa* in comparison with modern races. *Environ. Microbiol.* 2 131–141. 10.1046/j.1462-2920.2000.00078.x 11220300

[B43] Erisman J. W. Sutton M. A. Galloway J. Klimont Z. Winiwarter W. (2008). How a century of ammonia synthesis changed the world. *Nat. Geosci.* 1 636–639. 10.1038/ngeo325

[B44] Estrada-de los Santos P. Vinuesa P. Martínez-Aguilar L. Hirch A. M. Caballero-Mellado J. (2013). Phylogenetics analysis of *Burkholderia* species by multilocus sequence analysis. *Curr. Microbiol.* 67 51–60. 10.1007/s00284-013-0330-9 23404651

[B45] Fiore A. Laevens S. Bevivino A. Dalmastric C. Tabacchioni S. Vandamme P. (2001). *Burkholderia cepacia* complex: distribution of genomovars among isolates from the maize rhizosphere in Italy. *Environ. Microbiol.* 3 137–143. 10.1046/j.1462-2920.2001.00175.x 11321544

[B46] Fischer D. Pfitzner B. Schmid M. Simões-Araújo J. L. Reis V. M. Pereira W. (2012). Molecular characterization of the diazotrophic bacterial community in uninoculated and inoculated field-grown sugarcane (*Saccharum* sp.). *Plant Soil* 356 83–99. 10.1007/s11104-011-0812-0

[B47] Fouts D. E. Tyler H. L. DeBoy R. T. Daugherty S. Ren Q. Badger J. H. (2008). Complete genome sequence of the N_2_-fixing broad host range endophyte *Klebsiella pneumoniae* 342 and virulence predictions verified in mice. *PLoS Genet.* 4:e1000141. 10.1371/journal.pgen.1000141 18654632 PMC2453333

[B48] Fox A. R. Soto G. Valverde C. Russo D. Lagares A. Jr. Zorreguieta A. (2016). Major cereal crops benefit from biological nitrogen fixation when inoculated with the nitrogen-fixing bacterium *Pseudomonas protegens* Pf-5 X940. *Environ. Microbiol.* 18 3522–3534. 10.1111/1462-2920.13376 27198923

[B49] Friesen M. L. Porter S. S. Stark S. C. von Wettberg E. J. Sachs J. L. Martínez-Romero E. (2011). Microbially mediated plant functional traits. *Annu. Rev. Ecol. Evol. Syst.* 42 23–46. 10.1146/annurev-ecolsys-102710-145039

[B50] Fürnkranz M. Lukesch B. Müller H. Huss H. Grube M. Berg G. (2012). Microbial diversity inside pumpkins: microhabitat-specific communities display a high antagonistic potential against phytopathogens. *Microb. Ecol* 63 418–428. 10.1007/s00248-011-9942-4 21947430

[B51] Geddes B. A. Ryu M. H. Mus F. Garcia Costas A. Peters J. W. Voigt C. A. (2015). Use of plant colonizing bacteria as chassis for transfer of N2-fixation to cereals. *Curr. Opin. Biotechnol.* 32 216–222. 10.1016/j.copbio.2015.01.004 25626166

[B52] Gibson A. H. (1966). The carbohydrate requirement for symbiotic nitrogen fixation: a “whole plant” growth analysis approach. *Aust. J. Biol. Sci.* 19 499–515. 10.1071/BI9660499

[B53] Gillis M. Tran Van V. Bardin R. Goor M. Hebbar P. Willems A. (1995). Polyphasic taxonomy in the genus *Burkholderia* leading to an amended description of the genus and proposition of *Burkholderia vietnamiensis* sp. Nov. for N_2_-fixing isolates from rice in Vietnam. *Int. J. Syst. Bacteriol.* 45 274–289. 10.1099/00207713-45-2-274

[B54] Gomes N. C. M. Heuer H. Schönfeld J. Costa R. Mendonça-Hagler L. Smalla K. (2001). Bacterial diversity of the rhizosphere of maize (*Zea mays*) grown in tropical soil studied by temperature gradient gel electrophoresis. *Plant Soil* 232 167–180. 10.1023/A:1010350406708

[B55] González-Pasayo R. Martínez-Romero E. (2000). Multiresistance genes of *Rhizobium etli* CFN42. *Mol. Plant Microbe Interact.* 13 572–577. 10.1094/MPMI.2000.13.5.572 10796024

[B56] Govedarica M. (1990). Specific relationship between *Beijerinckia* Derx strains and some maize hybrids. *Zemljište Biljka* 39 125–132.

[B57] Govindarajan M. Balandreau J. Muthukumarasamy R. Revathi G. Lakshminarasimhan C. (2006). Improved yield of micropropagated sugarcane following inoculation by endophytic *Burkholderia vietnamiensis*. *Plant Soil* 280 239–252. 10.1007/s11104-005-3223-2

[B58] Guntli D. Heeb M. Moënne-Loccoz Y. Défago G. (1999). Contribution of calystegine catabolic plasmid to competitive colonization of the rhizosphere of calystegine-producing plants by *Sinorhizobium meliloti* Rm41. *Mol. Ecol.* 8 855–863. 10.1046/j.1365-294X.1999.00640.x

[B59] Guo B. Wang Y. Sun X. Tang K. (2008). Bioactive natural products from endophytes: a review. *Prikl. Biokhim. Mikrobiol.* 44 153–158. 10.1134/S0003683808020026 18669256

[B60] Gutiérrez-Zamora M. L. Martínez-Romero E. (2001). Natural endophytic association between *Rhizobium etli* and maize (*Zea mays* L.). *J. Biotechnol.* 91 117–126. 10.1016/s0168-1656(01)00332-7 11566384

[B61] Gutjahr C. Banba M. Croset V. An K. Miyao A. An G. (2008). Arbuscular mycorrhiza–specific signaling in rice transcends the common symbiosis signaling pathway. *Plant Cell* 20 2989–3005. 10.1105/tpc.108.062414 19033527 PMC2613669

[B62] Haichar F. Z. Marol C. Berge O. Rangel-Castro J. I. Prosser J. I. Balesdent J. (2008). Plant host habitat and root exudates shape soil bacterial community structure. *ISME J.* 2 1221–1230. 10.1038/ismej.2008.80 18754043

[B63] Han J. Song Y. Liu Z. Hu Y. (2011). Culturable bacterial community analysis in the root domains of two varieties of tree peony (*Paeonia ostii*). *FEMS Microbiol. Lett.* 322 15–24. 10.1111/j.1574-6968.2011.02319.x 21623895

[B64] Hardoim P. R. Hardoim C. C. P. van Overbeek L. S. van Elsas J. D. (2012). Dynamics of seed-borne rice endophytes on early plant growth stages. *PLoS One* 7:e30438. 10.1371/journal.pone.0030438 22363438 PMC3281832

[B65] Hartmann A. Schmid M. van Tuinen D. Berg G. (2009). Plant-driven selection of microbes. *Plant Soil* 321 235–257. 10.1007/s11104-008-9814-y

[B66] Hurek T. Handley L. L. Reinhold-Hurek B. Piché Y. (2002). *Azoarcus* grass endophytes contribute fixed nitrogen to the plant in an unculturable state. *Mol. Plant Microbe Interact.* 15 233–242. 10.1094/MPMI.2002.15.3.233 11952126

[B67] Isobe K. Ohte N. (2014). Ecological perspectives on microbes involved in N-cycling. *Microbes Environ.* 29 4–16. 10.1264/jsme2.ME13159 24621510 PMC4041230

[B68] Ivleva N. B. Groat J. Staub J. M. Stephens M. (2016). Expression of active subunit of nitrogenase via integration into plant organelle genome. *PLoS One* 11:e0160951. 10.1371/journal.pone.0160951 27529475 PMC4986947

[B69] James E. K. Gyaneshwar P. Mathan N. Barraquio W. L. Reddy P. M. Iannetta P. P. (2002). Infection and colonization of rice seedlings by the plant growth-promoting bacterium *Herbaspirillum seropedicae* Z67. *Mol. Plant Microbe Interact.* 15 894–906. 10.1094/MPMI.2002.15.9.894 12236596

[B70] Johnston-Monje D. Mousa W. K. Lazarovits G. Raizada M. N. (2014). Impact of swapping soils on the endophytic bacterial communities of pre-domesticated, ancient and modern maize. *BMC Plant Biol.* 14:233. 10.1186/s12870-014-0233-3 25227492 PMC4189167

[B71] Johnston-Monje D. Raizada M. N. (2011). Conservation and diversity of seed associated endophytes in *Zea* across boundaries of evolution, ethnography and ecology. *PLoS One* 6:e20396. 10.1371/journal.pone.0020396 21673982 PMC3108599

[B72] Kennedy I. R. Choudhury A. T. M. A. Kecskés M. L. (2004). Non-symbiotic bacterial diazotrophs in crop-farming systems: can their potential for plant growth promotion be better exploited? *Soil Biol. Biochem.* 36 1229–1244. 10.1016/j.soilbio.2004.04.006

[B73] Kennedy I. R. Cocking E. C. (1997). *Biological Nitrogen Fixation: The Global Challenge and Future Needs.* Sydney: SUNFix Press.

[B74] Khalaf E. M. Raizada M. N. (2016). Taxonomic and functional diversity of cultured seed associated microbes of the cucurbit family. *BMC Microbiol.* 16:131. 10.1186/s12866-016-0743-2 27349509 PMC4924336

[B75] Khush G. S. Bennett J. (eds) (1992). *Nodulation and Nitrogen Fixation in Rice: Potential and Prospects.* Manila: IRRI International Rice Research Institute.

[B76] Knief C. Delmotte N. Chaffron S. Stark M. Innerebner G. Wassmann R. (2012). Metaproteogenomic analysis of microbial communities in the phyllosphere and rhizosphere of rice. *ISME J.* 6 1378–1390. 10.1038/ismej.2011.192 22189496 PMC3379629

[B77] Krause A. Ramakumar A. Bartels D. Battistoni F. Bekel T. Boch J. (2007). Complete genome of the mutualistic N_2_-fixing grass endophyte *Azoarcus* sp. strain BH72. *Nat. Biotech.* 25:478. 10.1038/nbt1243 17057704

[B78] Kutter S. Hartmann A. Schmid M. (2006). Colonization of barley (*Hordeum vulgare*) with *Salmonella enterica* and *Listeria* spp. *FEMS Microbiol. Ecol.* 56 262–271. 10.1111/j.1574-6941.2005.00053.x 16629755

[B79] Ladha J. K. Reddy P. M. (eds) (2000). “The quest for nitrogen fixation in rice,” in *Proceedings of the 3rd Working Group Meeting on Assessing Opportunities for Nitrogen Fixation in Rice*, (Makati: International Rice Research Institute), 354.

[B80] Ladha J. K. Reddy P. M. (1995). Extension of nitrogen fixation to rice - Necessity and possibilities. *Geo J.* 35 363–372. 10.1007/BF00989144

[B81] Layzell D. B. (2000). “Carbon costs for endosymbiotic nitrogen fixation in legumes,” in *Proceedings of the 3rd Working Group Meeting on Assessing Opportunities for Nitrogen Fixation in Rice*, eds Ladha J. K. Reddy P. M. (Makati: International Rice Research Institute,), 341–342.

[B82] Lebeis S. L. Paredes S. H. Lundberg D. S. Breakfield N. Gehring J. McDonald M. (2015). Plant Microbiome. Salicylic acid modulates colonization of the root microbiome by specific bacterial taxa. *Science* 349 860–864. 10.1126/science.aaa8764 26184915

[B83] Li X. Rui J. Xiong J. Li J. He Z. Zhou J. (2014). Functional potential of soil microbial communities in the maize rhizosphere. *PLoS One* 9:e112609. 10.1371/journal.pone.0112609 25383887 PMC4226563

[B84] Liu H. Carvalhais L. C. Crawford M. Singh E. Dennis P. G. Pieterse C. M. J. (2017a). Inner plant values: diversity, colonization and benefits from endophytic bacteria. *Front. Microbiol.* 8:2552. 10.3389/fmicb.2017.02552 29312235 PMC5742157

[B85] Liu H. Zhang L. Meng A. Zhang J. Xie M. Qin Y. (2017b). Isolation and molecular identification of endophytic diazotrophs from seeds stems of three cereal crops. *PLoS One* 12:e0187383. 10.1371/journal.pone.0187383 29084254 PMC5662235

[B86] López J. L. Alvarez F. Príncipe A. Salas M. E. Lozano M. J. Draghi W. O. (2018). Isolation, taxonomic analysis, and phenotypic characterization of bacterial endophytes present in alfalfa (*Medicago sativa*) seeds. *J. Biotechnol.* 267 55–62. 10.1016/j.jbiotec.2017.12.020 29292130

[B87] López-Guerrero M. Ramírez M. A. Martínez-Romero E. (2013). “Rhizobial genetic repertoire to inhabit legume and nonlegume rhizospheres,” in *Molecular Microbial Ecology of the Rhizosphere*, Vol. 1 2 ed. Bruijn F. J. de (Hoboken, NJ: John Wiley & Sons, Inc), 10.1002/9781118297674.ch46

[B88] López-López A. Negrete-Yankelevich S. Rogel M. A. Ormeño-Orrillo E. Martínez J. Martínez-Romero E. (2012). Native bradyrhizobia from Los Tuxtlas in Mexico are symbionts of *Phaseolus lunatus* (Lima bean). *Syst. Appl. Microbiol.* 36 33–38. 10.1016/j.syapm.2012.10.006 23280323

[B89] López-Torrejón G. Jiménez-Vicente E. Buesa J. M. Hernandez J. A. Verma H. K. Rubio L. M. (2016). Expression of a functional oxygen-labile nitrogenase component in the mitochondrial matrix of aerobically grown yeast. *Nat. Commun.* 7:11426. 10.1038/ncomms11426 27126134 PMC4855529

[B90] Lundberg D. S. Lebeis S. L. Paredes S. H. Yourstone S. Gehring J. Malfatti S. (2012). Defining the core *Arabidopsis thaliana* root microbiome. *Nature* 488 86–90. 10.1038/nature11237 22859206 PMC4074413

[B91] Lynch J. M. Whipps J. M. (1990). Substrate flow in the rhizosphere. *Plant Soil* 129 1–10. 10.1007/BF00011685

[B92] Madmony A. Chernin L. Pleban S. Peleg E. Riov J. (2005). *Enterobacter cloacae*, an obligatory endophyte of pollen grains of Mediterranean pines. *Folia Microbiol.* 50 209–216. 10.1007/BF02931568 16295659

[B93] Markmann K. Parniske M. (2008). Evolution of root endosymbiosis with bacteria: How novel are nodules? *Trends Plant Sci.* 14 77–86. 10.1016/j.tplants.2008.11.009 19167260

[B94] Mårtensson L. Díez B. Wartiainen I. Zheng W. El-Shehawy R. Rasmussen U. (2009). Diazotrophic diversity, *nifH* gene expression and nitrogenase activity in a rice paddy field in Fujian, China. *Plant Soil* 325 207–218. 10.1007/s11104-009-9970-8

[B95] Martínez J. Martínez L. Rosenblueth M. Silva J. Martínez-Romero E. (2004). How are gene sequence analyses modifying bacterial taxonomy? The case of *Klebsiella*. *Int. Microbiol.* 7 261–268. 15666246

[B96] Martínez L. Caballero J. Orozco J. Martínez-Romero E. (2003). Diazotrophic bacteria associated with banana (*Musa* spp.). *Plant Soil* 257 35–47. 10.1023/A:1026283311770

[B97] Martínez-Romero E. Rodríguez-Medina N. Beltrán-Rojel M. Toribio-Jiménez J. Garza-Ramos U. (2018). *Klebsiella variicola* and *Klebsiella quasipneumoniae* with capacity to adapt to clinical and plant settings. *Salud Pública Méx.* 60 29–40. 10.21149/8156 29689654

[B98] Martínez-Romero E. Silva-Sanchez J. Barrios H. Rodríguez-Medina N. Martínez-Barnetche J. Téllez-Sosa J. (2015). Draft genome sequences of *Klebsiella variicola* plant isolates. *Genome Announc.* 3:e01015-15. 10.1128/genomeA.01015-15 PMC4566181 26358599

[B99] Matiru V. N. Dakora F. D. (2004). Potential use of rhizobial bacteria as promoters of plant growth for increased yield in landraces of African cereal crops. *African J. Biotechnol.* 3 1–7. 10.5897/AJB2004.000-2002

[B100] Mendes R. Garbeva P. Raaijmakers J. M. (2013). The rhizosphere microbiome: significance of plant beneficial, plant pathogenic, and human pathogenic microorganisms. *FEMS Microbiol. Rev.* 37 634–663. 10.1111/1574-6976.12028 23790204

[B101] Mendes R. Pizzirani-Kleiner A. A. Araujo W. L. Raaijmakers J. M. (2007). Diversity of cultivated endophytic bacteria from sugarcane: genetic and biochemical characterization of *Burkholderia cepacia* complex isolates. *Appl. Environ. Microbiol.* 72 7259–7267. 10.1128/AEM.01222-07 17905875 PMC2168197

[B102] Merino-Flores M. (2012). *Diversidad Bacteriana de la Rizósfera de Maíz Regado Con Agua Del Río Apatlaco Mediante un Enfoque Independiente De Cultivo (Búsqueda de Micobacterias).* Ph.D. thesis, Universidad Autónoma del Estado de Morelos, Cuernavaca.

[B103] Mitter B. Pfaffenbichler N. Flavell R. Compant S. Antonielli L. Petric A. (2017). A New approach to modify plant microbiomes and traits by introducing beneficial bacteria at flowering into progeny seeds. *Front. Microbiol.* 8:11. 10.3389/fmicb.2017.00011 28167932 PMC5253360

[B104] Molina M. A. Ramos J. L. Espinosa-Urgel M. (2006). A two-partner secretion system is involved in seed and root colonization and iron uptake by *Pseudomonas putida* KT2440. *Environ. Microbiol.* 8 639–647. 10.1111/j.1462-2920.2005.00940.x 16584475

[B105] Morris J. J. Lenski R. E. Zinser E. R. (2012). The black queen hypothesis: evolution of dependencies through adaptive gene loss. *mBio.* 3:e00036-12. 10.1128/mBio.00036-12 22448042 PMC3315703

[B106] Mus F. Crook M. B. Garcia K. Garcia Costas A. Geddes B. A. Kouri E. D. (2016). Symbiotic nitrogen fixation and the challenges to its extension to nonlegumes. *Appl. Environ. Microbiol.* 82 3698–3710. 10.1128/AEM.01055-16 27084023 PMC4907175

[B107] Muthukumarasamy R. Cleenwerck I. Revathi G. Vadivelu M. Janssens D. Hoste B. (2005). Natural association of *Gluconacetobacter diazotrophicus* and diazotrophic *Acetobacter peroxydans* with wetland rice. *Syst. Appl. Microbiol.* 28 277–286. 10.1016/j.syapm.2005.01.006 15900973

[B108] Okon Y. Itzigsohn R. (1995). The development of *Azospirillum* as a commercial inoculant for improving crop yields. *Biotechnol. Adv.* 13 415–424. 10.1016/0734-9750(95)02004-M 14536095

[B109] Okunishi S. Sako K. Mano H. Imamura A. Morisaki H. (2005). Bacterial flora of endophytes in the maturing seed of cultivated rice (*Oryza sativa*). *Microbes Environ.* 20 168–177. 10.1264/jsme2.20.168

[B110] Ormeño-Orrillo E. Hungria M. Martínez-Romero E. (2013). “Dinitrogen-fixing prokaryotes,” in *The Prokaryotes: Prokaryotic Physiology and Biochemistry*, eds Rosenberg E. Long E. F. de Lory S. Stackebrandt E. Thompson F. (Berlin: Springer-Verlag), 427–451. 10.1007/978-3-642-30141-4_72

[B111] Pankievicz V. C. Camilios-Neto D. Bonato P. Balsanelli E. Tadra-Sfeir M. Z. Faoro H. (2016). RNA-seq transcriptional profiling of *Herbaspirillum seropedicae* colonizing wheat (*Triticum aestivum*) roots. *Plant Mol. Biol.* 90 589–603. 10.1007/s11103-016-0430-6 26801330

[B112] Parniske M. Ahlborn B. Werner D. (1991). Isoflavonoid-inducible resistance to the phytoalexin glyceollin in soybean rhizobia. *J. Bacteriol.* 173 3432–3439. 10.1128/jb.173.11.3432-3439.1991 2045365 PMC207956

[B113] Pate J. S. Layzell D. B. Atkins C. A. (1979). Economy of carbon and nitrogen in a nodulated and non-nodulated (NO_3_-grown) legume. *Plant Physiol.* 64 1083–1088. 10.1104/pp.64.6.1083 16661097 PMC543196

[B114] Paul M. J. Foyer C. H. (2001). Sink regulation of photosynthesis. *J. Exp. Bot.* 52 1383–1400. 10.1093/jexbot/52.360.1383 11457898

[B115] Pedrosa F. O. Monteiro R. A. Wassem R. Cruz L. M. Ayub R. A. Colauto N. B. (2011). Genome of *Herbaspirillum seropedicae* strain SmR1, a specialized diazotrophic endophyte of tropical grass. *PLoS Gentet.* 7:e1002064. 10.1371/journal.pgen.1002064 21589895 PMC3093359

[B116] Peiffer J. A. Spor A. Koren O. Jin Z. Tringe S. G. Dangl J. L. (2013). Diversity and heritability of the maize rhizosphere microbiome under field conditions. *Proc. Natl. Acad. Sci. U.S.A.* 110 6548–6553. 10.1073/pnas.1302837110 23576752 PMC3631645

[B117] Peralta H. Aguilar A. Díaz R. Mora Y. Martínez-Batallar G. Salazar E. (2016). Genomic studies of nitrogen-fixing rhizobial strains from *Phaseolus vulgaris* seeds and nodules. *BMC Genomics* 17:711. 10.1186/s12864-016-3053-z 27601031 PMC5011921

[B118] Pereira P. Ibáñez F. Rosenblueth M. Etcheverry M. Martínez-Romero E. (2011). Analysis of the bacterial diversity associated with the roots of maize (*Zea mays* L.) by culture dependent and culture independent methods. *ISRN Ecol.* 2011:938546 10.5402/2011/938546

[B119] Perin L. Martínez-Aguilar L. Castro-González R. Estrada-de Los Santos P. Cabellos-Avelar T. Guedes H. V. (2006). Diazotrophic *Burkholderia* species associated with field-grown maize and sugarcane. *Appl. Environ. Microbiol.* 72 3103–3110. 10.1128/AEM.72.5.3103-3110.2006 16672447 PMC1472400

[B120] Pfeiffer B. Fender A.-C. Lasota S. Hertel D. Jungkunst H. F. Daniel R. (2013). Leaf litter is the main driver for changes in bacterial community structures in the rhizosphere of ash and beech. *Appl. Soil Ecol.* 72 150–160. 10.1016/j.apsoil.2013.06.008

[B121] Pfeiffer S. Mitter B. Oswald A. Schloter-Hai B. Schloter M. Declerck S. (2017). Rhizosphere microbiomes of potato cultivated in the High Andes show stable and dynamic core microbiomes with different responses to plant development. *FEMS Microbiol. Ecol.* 93:fiw242. 10.1093/femsec/fiw242 27940644

[B122] Pracht J. E. Nickell C. D. Harper J. E. Bullock D. G. (1994). Agronomic evaluation of non-nodulating and hypernodulating mutants of soybean. *Crop Sci.* 34 738–740. 10.2135/cropsci1994.0011183X003400030025x

[B123] Rangjaroen C. Rerkasem B. Teaumroong N. Sungthong R. Lumyong S. (2014). Comparative study of endophytic and endophytic diazotrophic bacterial communities across rice landraces grown in the highlands of northern Thailand. *Arch. Microbiol.* 196 35–49. 10.1007/s00203-013-0940-4 24264469

[B124] Ray D. K. Mueller N. D. West P. C. Foley J. A. (2013). Yield trends are insufficient to double global crop production by 2050. *PLoS One* 8:e66428. 10.1371/journal.pone.0066428 23840465 PMC3686737

[B125] Reddy P. M. Altúzar-Molina A. R. Ortiz-Berrocal M. Medina-Andrés R. López-Sámano M. Martínez-Aguilar L. (2013). “Predisposition and redesigning of genetic networks of rice for accommodating nitrogen-fixing rhizobial symbiosis,” in *International Dialogue on Perception and Prospects of Designer Rice*, eds Muralidharan K. Siddiq E. A. (Hyderabad: Society for Advancement of Rice Research), 245–257.

[B126] Reddy P. M. James E. K. Ladha J. K. (2002). “Nitrogen fixation in rice,” in *Nitrogen Fixation at the Millennium*, ed. Leigh G. J. (Amsterdam: Elsevier), 421–445. 10.1016/B978-044450965-9/50015-X

[B127] Reinhold-Hurek B. Hurek T. (1997). *Azoarcus* spp. and their interactions with grass roots. *Plant Soil* 194 57–64. 10.1023/A:1004216507507

[B128] Reyna-Flores F. Barrios-Camacho H. Dantán-González E. Ramírez-Trujillo J. A. Lozano Aguirre Beltrán L. F. Rodríguez-Medina N. (2018). Draft genome sequences of endophytic isolates of *Klebsiella variicola* and *Klebsiella pneumoniae* obtained from the same sugarcane plant. *Genome Announc.* 6:e00147-18. 10.1128/genomeA.00147-18 29567733 PMC5864947

[B129] Rijavec T. Lapange A. Dermastia M. Rupnik M. (2007). Isolation of bacterial endophytes from germinated maize kernels. *Can. J. Microbiol.* 53 802–808. 10.1139/W07-048 17668041

[B130] Roesch L. F. W. Camargo F. A. O. Bento F. M. Triplett E. W. (2008). Biodiversity of diazotrophic bacteria within the soil, root and stem of field-grown maize. *Plant Soil* 302 91–104. 10.1007/s11104-007-9458-3

[B131] Rogers C. Oldroyd G. E. D. (2014). Synthetic biology approaches to engineering the nitrogen symbiosis in cereals. *J. Exp. Bot.* 65 1939–1946. 10.1093/jxb/eru098 24687978

[B132] Roncato-Maccari L. D. Ramos H. J. Pedrosa F. O. Alquini Y. Chubatsu L. S. Yates M. G. (2003). Endophytic *Herbaspirillum seropedicae* expresses *nif* genes in gramineous plants. *FEMS Microbiol. Ecol.* 45 39–47. 10.1016/S0168-6496(03)00108-9 19719605

[B133] Rosenblueth M. López-López A. Martínez J. Rogel M. A. Toledo I. Martínez Romero E. (2012). Seed bacterial endophytes: common genera, seed to seed variability and possible roles in plants. *Acta Hortic.* 938 39–48. 10.17660/ActaHortic.2012.938.4

[B134] Rosenblueth M. Martínez L. Silva J. Martínez-Romero E. (2004). *Klebsiella variicola*, a novel species with clinical and plant-associated isolates. *Syst. Appl. Microbiol.* 27 27–35. 10.1078/0723-2020-00261 15053318

[B135] Rosenblueth M. Martínez-Romero E. (2004). *Rhizobium etli* maize populations and their competitiveness for root colonization. *Arch. Microbiol.* 181 337–344. 10.1007/s00203-004-0661-9 15024554

[B136] Rosenblueth M. Martínez-Romero E. (2006). Bacterial endophytes and their interactions with hosts. *Mol. Plant Microbe Interact.* 19 827–837. 10.1094/MPMI-19-0827 16903349

[B137] Rosenblueth M. Martínez-Romero J. C. Reyes-Prieto M. Rogel M. A. Martínez-Romero E. (2011). Environmental mycobacteria: a threat to human health? *DNA Cell Biol.* 30 633–640. 10.1089/dna.2011.1231 21595554

[B138] Rossbach S. McSpadden B. Kulpa D. Rasul G. Ganoof M. de Bruijn F. J. (1994). Use of rhizopine synthesis and catabolism genes to monitor soil bacteria and to create biased rhizospheres. *Mol. Ecol.* 3 610–611.

[B139] Sachman-Ruiz B. Castillo-Rodal A. I. López-Vidal Y. Martínez-Romero E. Vinuesa P. (2009). “Diversity of environmental mycobacteria in Mexican rivers assessed by cultivation and metagenomic approaches,” in *Proceedings of the 109th General Meeting*, Philadelphia, PA, 17–21.

[B140] Saharan B. Nehra V. (2011). Plant growth promoting rhizobacteria: a critical review. *Life Sci. Med. Res.* 21 1–30.

[B141] Santi C. Bogusz D. Franche C. (2013). Biological nitrogen fixation in non-legume plants. *Ann. Bot.* 111 743–767. 10.1093/aob/mct048 23478942 PMC3631332

[B142] Sawada S. Hayakawa T. Fukushi K. Kasai M. (1986). Influence of carbohydrates on photosynthesis in single, rooted soybean leaves used as a source–sink model. *Plant Cell Physiol.* 27 591–600.

[B143] Sawana A. Adeolu M. Gupta R. S. (2014). Molecular signatures and phylogenomics analysis of the genus *Burkholderia*: proposal for division of this genus into the emended genus *Burkholderia* containing pathogenic organisms and a new genus *Paraburkholderia* gen. nov. harboring environmental species. *Front. Genet.* 5:429 10.3389/fgene.2014.00429 PMC4271702 25566316

[B144] Schmalenberger A. Tebbe C. C. (2003). Bacterial diversity in maize rhizospheres: conclusions on the use of genetic profiles based on PCR-amplified partial small subunit rRNA genes in ecological studies. *Mol. Ecol.* 12 251–262. 10.1046/j.1365-294X.2003.01716.x 12492893

[B145] Setten L. Soto G. Mozzicafreddo M. Fox A. R. Lisi C. Cuccioloni M. (2013). Engineering *Pseudomonas protegens* Pf-5 for nitrogen fixation and its application to improve plant growth under nitrogen-deficient conditions. *PLoS One* 8:e63666. 10.1371/journal.pone.0063666 23675499 PMC3652814

[B146] Shahzad R. Waqas M. Khan A. L. Asaf S. Khan M. A. Kang S. M. (2016). Seed-borne endophytic *Bacillus amyloliquefaciens* RWL-1 produces gibberellins and regulates endogenous phytohormones of *Oryza sativa*. *Plant Physiol. Biochem.* 106 236–243. 10.1016/j.plaphy.2016.05.006 27182958

[B147] Sharkey T. D. Stitt M. Heineke D. Gerhardt R. Raschke K. Heldt H. W. (1986). Limitation of photosynthesis by carbon metabolism: II. O_2_-insensitive CO_2_ uptake results from limitation of triose phosphate utilization. *Plant Physiol.* 81 1123–1129. 10.1104/pp.81.4.1123 16664954 PMC1075496

[B148] Smil V. (2002). Nitrogen and food production: proteins for human diets. *Ambio* 31 126–131. 10.1579/0044-7447-31.2.126 12078001

[B149] Song L. Carroll B. J. Gresshoff P. M. Herridge D. F. (1995). Field assessment of supernodulating genotypes of soybean for yield, N_2_ fixation and benefit to subsequent crops. *Soil Biol. Biochem.* 27 563–569. 10.1016/0038-0717(95)98632-X

[B150] Stacey G. McAlvin C. B. Kim S. Y. Olivares J. Soto M. J. (2006). Effects of endogenous salicylic acid on nodulation in the model legumes *Lotus japonicus* and *Medicago truncatula*. *Plant Physiol.* 141 1473–1481. 10.1104/pp.106.080986 16798946 PMC1533935

[B151] Stevenson F. J. Cole M. A. (1999). *Cycles of Soils: Carbon, Nitrogen, Phosphorus, Sulfur, Micronutrients.* Hoboken, NJ: John Wiley & Sons.

[B152] Stokstad E. (2016). The nitrogen fix. *Science* 353 1225–1227. 10.1126/science.353.6305.1225 27634521

[B153] Terakado-Tonooka J. Ohwaki Y. Yamakawa H. Tanaka F. Yoneyama T. Fujihara S. (2008). Expressed *nifH* genes of endophytic bacteria detected in field-grown sweet potatoes (*Ipomoea batatas* L.). *Microbes Environ.* 23 89–93. 10.1264/jsme2.23.89 21558693

[B154] Thaweenut N. Hachisuka Y. Ando S. Yanagisawa S. Yoneyama T. (2011). Two seasons’ study on nifH gene expression and nitrogen fixation by diazotrophic endophytes in sugarcane (*Saccharum* spp. hybrids): expression of *nifH* genes similar to those of rhizobia. *Plant Soil* 338 435–449. 10.1007/s11104-010-0557-1

[B155] Triplett E. W. (1996). Diazotrophic endophytes: progress and prospects for nitrogen fixation in monocots. *Plant Soil* 186 29–38. 10.1007/BF00035052

[B156] Ueda T. Suga Y. Yahiro N. Matsuguchi T. (1995). Remarable N_2_-fixing bacterial diversity detected in rice roots by molecular evolutionary analysis of *nifH* gene sequences. *J. Bacteriol.* 177 1414–1417. 10.1128/jb.177.5.1414-1417.1995 7868622 PMC176754

[B157] Van Dommelen A. Croonenborghs A. Spaepen S. Vanderleyden J. (2009). Wheat growth promotion through inoculation with an ammonium-excreting mutant of *Azospirillum brasilense*. *Biol. Fertil. Soils* 45 549–553. 10.1007/s00374-009-0357-z

[B158] Vance C. P. Heichel G. H. (1991). Carbon in N_2_ fixation: limitation or exquisite adaptation. *Annu. Rev. Plant Physiol. Plant Mol. Biol.* 42 373–390. 10.1146/annurev.pp.42.060191.002105

[B159] Venturi V. Keel C. (2016). Signaling in the rhizosphere. *Trends Plant Sci.* 21 187–198. 10.1016/j.tplants.2016.01.005 26832945

[B160] Verma S. K. Kingsley K. Irizarry I. Bergen M. Kharwar R. N. White J. F. Jr. (2017). Seed-vectored endophytic bacteria modulate development of rice seedlings. *J. Appl. Microbiol.* 122 1680–1691. 10.1111/jam.13463 28375579

[B161] Vermeiren H. Willems A. Schoofs G. de Mot R. Keijers V. Hai W. (1999). The rice inoculant strain *Alcaligenes faecalis* A15 is a nitrogen-fixing *Pseudomonas stutzeri*. *Syst. Appl. Microbiol.* 22 215–224. 10.1016/S0723-2020(99)80068-X 10390872

[B162] Wagner M. R. Lundberg D. S. Del Rio T. G. Tringe S. G. Dangl J. L. Mitchell-Olds T. (2016). Host genotype and age shape the leaf and root microbiomes of a wild perennial plant. *Nat. Commun.* 7:12151. 10.1038/ncomms12151 27402057 PMC4945892

[B163] Wang W. Zhai Y. Cao L. Tan H. Zhang R. (2016). Illumina-based analysis of core actinobacteriome in roots, stems, and grains of rice. *Microbiol. Res.* 190 12–18. 10.1016/j.micres.2016.05.003 27393994

[B164] Wartiainen I. Eriksson T. Zheng W. Rasmussen U. (2008). Variation in the active diazotrophic community in rice paddy–*nifH* PCR-DGGE analysis of rhizosphere and bulk soil. *Appl. Soil Ecol.* 39 65–75. 10.1016/j.apsoil.2007.11.008

[B165] Xiaoe Y. Xi S. (1991). Physiological effect of nitrate or ammonia top-dressing on hybrid and conventional rice varieties at late growth stage. *Acta Agron. Sin.* 17 283–291.

[B166] Yanni Y. G. Rizk R. Y. Corich V. Squartini A. Ninke K. Philip-Hollingsworth S. (1997). Natural endophytic association between *Rhizobium leguminosarum* bv. trifolii and rice roots and assessment of its potential to promote rice growth. *Plant Soil* 194 99–114. 10.1023/A:1004269902246

[B167] You M. Nishiguchi T. Saito A. Isawa T. Mitsui H. Minamisawa K. (2005). Expression of the *nifH* gene of a *Herbaspirillum* endophyte in wild rice species: daily rhythm during the light-dark cycle. *Appl. Environ. Microbiol.* 71 8183–8190. 10.1128/AEM.71.12.8183-8190.2005 16332801 PMC1317309

[B168] Zawoznik M. S. Vázquez S. C. Díaz Herrera S. M. Groppa M. D. (2014). Search for endophytic diazotrophs in barley seeds. *Braz. J. Microbiol.* 45 621–625. 10.1590/S1517-83822014000200033 25242949 PMC4166290

[B169] Zhang T. Yan Y. He S. Ping S. Alam K. M. Han Y. (2012). Involvement of the ammonium transporter AmtB in nitrogenase regulation and ammonium excretion in *Pseudomonas stutzeri* A1501. *Res. Microbiol.* 163 332–339. 10.1016/j.resmic.2012.05.002 22659337

